# PBDE Flame Retardants and Thyroid Hormones during Pregnancy

**DOI:** 10.1289/ehp.1002782

**Published:** 2010-12

**Authors:** Julie E. Goodman, Laura E. Kerper, Giffe T. Johnson, Raymond D. Harbison, Rocio Cordero, Richard V. Lee, Milo F. Pulde, Marcia Hardy, Todd Stedeford

**Affiliations:** Gradient, Cambridge, Massachusetts, E-mail: jgoodman@gradientcorp.com; Center for Environmental and Occupational Risk Analysis and Management, College of Public Health, University of South Florida, Tampa, Florida; Division of Endocrinology, University of Texas Medical School Houston, Texas; Department of Medicine and Obstetrics, State University of New York, Buffalo, New York; Brigham and Women’s Hospital and Harvard Medical School, Harvard University, Boston Massachusetts; Health, Safety & Environment, Albemarle Corporation, Baton Rouge, Louisiana

Chevrier et al. (2010) assessed the association between 10 polybrominated diphenylethers (PBDEs) and free and total thyroxine (T_4_) and thyroid-stimulating hormone (TSH) in 270 women around the 27th week of gestation. They concluded that PBDEs are associated with lower TSH levels during pregnancy, but several factors that likely influenced the results were not considered in their analysis.

Normal pregnancy can lead to low TSH levels without affecting T_4_ levels, as can several other factors (e.g., starvation; stress; psychiatric disorders; depression; acute or chronic nonthyroidal disorders; alterations in thyrotropin- releasing hormone, cortisol, opiodergic, dopaminergic, or somatostatinergic activity; and alterations in leptin and cytokine production) (Braverman and Utiger 2000; Krassas et al. 2010). None of these were included in the analyses of Chevrier et al. (2010), yet any of them could have contributed to lower TSH levels.

Chevrier et al. (2010) classified women in their second and third trimesters as having subclinical hyperthyroidism if their serum TSH levels were < 0.5 mIU/L and < 0.8 mIU/L, respectively. Krassas et al. (2010) recently reported TSH reference ranges: 5th percentiles for the second and third trimesters were 0.03–0.39 and 0.13 mIU/L, respectively, suggesting that the cutoffs used by Chevrier et al. (2010) led to an incorrect classification of at least some women.

Chevrier et al. (2010) reported that blood was drawn at 27.3 ± 3.1 (mean ± SD) weeks gestation; however, it is unclear which women were in the second trimester and which were in the third trimester. Although Chevrier et al. (2010) adjusted for gestational age, the use of different cutoffs for a binary variable (subclinical hyperthyroidism) for women in the same analyses likely biased results, particularly considering that differences between women near the end of the second and beginning of the third trimester are not great.

With the exception of a few outliers, the range of each PBDE among study subjects was quite small (all with the ratio of the 75th to the 25th percentile < 3.4). Because blood was drawn only once and all associations noted were quite weak, even a small difference between the measured PBDE level and the actual level in an individual could have biased the results.

All of the PBDE congeners were moderately to strongly intercorrelated (*r* = 0.6–0.9; *p* < 0.001), yet analyses were conducted only by individual congener, leaving the inappropriate impression that several of the PBDE congeners may have been causally associated with lower TSH levels.

The limitations discussed above preclude one from drawing conclusions regarding associations between serum PBDEs and TSH. It is notable, however, that even if associations are shown to be causal, the decrements in TSH reported are very small and mostly within the reference range for pregnant women. Thus, they are unlikely to result in adverse health effects in either pregnant women or their fetuses.
